# Gender-specific interactions of *MTHFR* C677T and *MTRR* A66G polymorphisms with overweight/obesity on serum lipid levels in a Chinese Han population

**DOI:** 10.1186/s12944-016-0354-9

**Published:** 2016-10-28

**Authors:** Xueyuan Zhi, Boyi Yang, Shujun Fan, Yanxun Wang, Jian Wei, Quanmei Zheng, Guifan Sun

**Affiliations:** 1 Research Center of Environment and Non-Communicable Disease, School of Public Health, China Medical University, No.77 Puhe Road, Shenyang North New Area, Shenyang, 110122 People’s Republic of China; 2 Guangzhou Key Laboratory of Environmental Pollution and Health Risk Assessment, Department of Preventive Medicine, School of Public Health, Sun Yat-Sen University, Guangzhou, 510080 China; 3 Division of Molecular Preventive Medicine, Shanghai Institute of Targeted Therapy and Molecular Medicine, Shanghai, 200433 China; 4 Brain Disease Center, Tianjin Dagang Oil Field General Hospital, Tianjin, 300280 China

**Keywords:** *MTHFR* C677T, *MTRR* A66G, Overweight, Obesity, Lipid, Interaction, Gender-specificity

## Abstract

**Background:**

Little is known regarding the interactions of methylenetetrahydrofolate reductase (*MTHFR*) C677T and methionine synthase reductase (*MTRR*) A66G polymorphisms with overweight/obesity on serum lipid profiles. The aim of the current study was to explore interactions between the two polymorphisms and overweight/obesity on four common lipid levels in a Chinese Han population and further to evaluate whether these interactions exhibit gender-specificity.

**Methods:**

A total of 2239 participants (750 females and 1489 males) were enrolled into this study. The genotypes of the *MTHFR* C677T and *MTRR* A66G were determined by a TaqMan assay. Overweight and obesity were defined as a body mass index between 24 and 27.99 and ≥ 28 kg/m^2^, respectively. The interactions were examined by factorial design covariance analysis, and further multiple comparisons were conducted by Bonferroni correction.

**Results:**

There was no significant difference in the genotypic and allelic frequencies between females and males (*MTHFR* 677 T allele: 54.47 % for females and 54.40 % for males; *MTRR* 66G allele: 24.73 % for females and 24.71 % for males). Interaction between the *MTHFR* C677T polymorphism and overweight/obesity on serum triglyceride levels, and interaction between the *MTRR* A66G polymorphism and overweight/obesity on serum high-density lipoprotein cholesterol levels were detected in women (*P* = 0.015 and *P* = 0.056, respectively). For female subjects with overweight/obesity, the serum triglyceride levels in *MTHFR* 677TT genotype [1.09 (0.78–1.50) mmol/L] were significantly higher as compared with *MTHFR* 677CC genotype [0.90 (0.60–1.15) mmol/L, *P* = 0.007], and the *MTRR* 66GG genotype carriers had higher serum high-density lipoprotein cholesterol levels than those with *MTRR* 66AG genotype (1.46 ± 0.50 vs. 1.19 ± 0.31 mmol/L, *P* = 0.058). Furthermore, in male subjects with overweight/obesity, the *MTHFR* 677CT genotype carriers had higher low-density lipoprotein cholesterol levels than those with *MTHFR* 677TT genotype (2.96 ± 1.07 vs. 2.74 ± 0.88 mmol/L, *P* = 0.015).

**Conclusions:**

Our results indicate that there exist interactive effects of the *MTHFR* C677T and *MTRR* A66G polymorphisms with overweight/obesity on some lipid traits in Chinese Han population, and the effects were gender-specific.

## Background

Dyslipidemia, serving as a crucial risk factor for cardiovascular diseases, has become a serious public health problem worldwide because of its high prevalence [1–3]. The etiology of dyslipidemia is complicated, and it is generally agreed that serum lipid concentrations are influenced by both genetic and environmental factors as well as their interactions [4–7].

Previous studies indicated that methylenetetrahydrofolate reductase (*MTHFR*) C677T and methionine synthase reductase (*MTRR*) A66G polymorphisms are significantly associated with serum lipid levels [8–11]. MTHFR catalyzes the reduction of methylenetetrahydrofolate to methyltetrahydrofolate, a key methyl donor for homocysteine remethylation to methionine [12]. At the 677 bp of *MTHFR* gene, a C to T switch (C677T) has been verified to reduce its enzyme activity, leading to accumulation of homocysteine [13–15]. MTRR is responsible for maintaining adequate levels of activated cobalamin, an indispensable cofactor for homocysteine remethylation. The *MTRR* A66G polymorphism results in difference in its enzyme expression and function, and further affects plasma homocysteine levels [16]. High homocysteine levels have been implicated to be associated with lipid change in numerous epidemiological studies [17–20]. Research with animal and cell models also demonstrated that high homocysteine concentrations interfere with some lipid parameters metabolism [21–24]. These links suggest that the *MTHFR* C677T and *MTRR* A66G polymorphisms may affect lipid concentrations by altering homocysteine levels.

However, not all prior studies have found significant correlations between the two polymorphisms and serum lipid levels. The inconsistency among studies may be partly due to different environmental factors that interact with gene to regulate serum lipid levels. Overweight/obesity is a well-established modifiable contributor to the development of dyslipidemia [25–27]. Research on the interactions of the *MTHFR* C677T and *MTRR* A66G polymorphisms with overweight/obesity may provide valuable insight into the inconsistency among prior studies. However, information on the interactions of the two polymorphisms and overweight/obesity on serum lipid profiles is scarce. In a systematic Medline search, we found only one related study. In that study, Yin et al. observed significant interactions between the *MTHFR* C677T polymorphism and overweight/obesity on some lipid traits in Chinese Bai Ku Yao population [5]. Whether this interaction still exists in Chinese Han population remains elusive.

In addition, gender differences in lipid and lipoprotein metabolism have been well documented [28–31]. However, the underlying mechanism is unclear. Despite some advances in the identification of sex-specific genetic association with plasma lipid levels [11, 32, 33], few studies have explored the modifying effects of sex in the interactions between single nucleotide polymorphisms (SNPs) and overweight/obesity on lipid concentrations. Such exploration may provide valuable insight into the factors causing gender difference in the plasma lipid profile.

This study, therefore, was conducted to explore the interactions of the *MTHFR* C677T and *MTRR* A66G polymorphisms with overweight/obesity on four common lipid traits in a Chinese Han population and further to assess whether these interactions exhibit gender-specificity.

## Methods

### Study population

From October 2008 to February 2011, a total of 2239 individuals (750 females, 33.50 % and 1489 males, 66.50 %) were recruited into our study from an unselected population taking a regular health examination at the physical examination center of Dagang Oil Field General Hospital. Subjects with a history of gastrointestinal, thyroid, chronic liver, renal diseases, or diabetes mellitus were excluded. None of these subjects were treated with a lipid-lowering diet or drugs known to affect serum lipid levels within 4 weeks before the study. There was no blood relationship among all the selected subjects, which were of Han nationality living in Dagang district in Tianjin. Age of the participants ranged from 22 to 78 years with the mean age of 45.38 ± 9.40 years in females and 47.37 ± 10.10 years in males, respectively.

### Clinical measurements and laboratory tests

National standard techniques were used to measure height and body weight, and body mass index (BMI) was calculated as weight in kilograms divided by the square of height in meters (Kg/m^2^). BMI < 24, 24–27.99 and ≥ 28 was classified as normal weight, overweight and obesity, respectively [34]. Blood pressure was measured three times while study subjects were in the sitting position after 15 min of rest and the three measurements were averaged for analysis. After an overnight fast, venous blood samples and buccal cell samples were collected from all the participants for biochemical analysis and genetic analysis, respectively. The levels of triglyceride (TG), total cholesterol (TC), high-density lipoprotein cholesterol (HDL-C), low-density lipoprotein cholesterol (LDL-C) and fasting blood glucose (FBG) in blood samples were measured by enzymatic method on a Hitachi Autoanalyzer (Type 7170A; Hitachi Ltd., Tokyo, Japan) in Dagang Oil Field General Hospital.

### Genotyping

Genomic DNA was extracted from buccal samples using the QIAamp DNA Mini Kit (Qiagen, Valencia, CA, USA). The genotypes of the *MTHFR* C677T and *MTRR* A66G were determined by a TaqMan assay, which has been described in our previous paper [35].

### Statistical analysis

All statistical analyses were carried out using SAS version 9.2 (SAS Institute, Cary, NC, USA) and two-sided *P* < 0.05 was considered to be statistically significant. Data were expressed as mean ± standard deviation or median (interquartile range) for quantitative variables and as *n* (%) for qualitative variables. Variables with skewed distribution (serum TG) were log transformed before analysis. Genotypic and allelic frequencies were calculated via direct counting. The distributions of genotypes were analyzed for deviation from Hardy-Weinberg equilibrium using Chi-square test. Comparisons of difference between the female and male subjects with respect to general characteristics and genotype distribution were performed by two-sample *t* test and Chi-square test, respectively (the difference in serum TG levels was determined by the Wilcoxon two-sample test). The interaction between overweight/obesity and two studied polymorphisms on serum lipid levels was tested by using a factorial design covariance analysis after controlling for age, and further multiple comparisons were conducted by Bonferroni correction.

## Results

### General characteristics and genotype distribution

Table 1 summarizes the basic characteristics and genotype distributions of the study population. Totally, 2239 participants were analyzed, of which 1489 (66.50 %) were male. Among the study population, females were significantly younger than males (*P* < 0.001). Compared with female subjects, the males had higher BMI and higher levels of systolic blood pressure, diastolic blood pressure, FBG, serum TG, TC and LDL-C (All *P* < 0.05), whereas the serum HDL-C levels was lower in men than in women (*P* < 0.001).

**Table 1:** General characteristics and genotype distribution

Variables	Total (*n* = 2239)	Female (*n* = 750)	Male (*n* = 1489)	*P*
Age, year	46.70 ± 9.92	45.38 ± 9.40	47.37 ± 10.10	<0.001
Height, cm	168.37 ± 7.67	161.80 ± 5.67	171.68 ± 6.28	<0.001
Weight, kg	71.25 ± 12.50	61.60 ± 9.50	76.11 ± 10.91	<0.001
BMI, kg/m^2^	25.03 ± 3.52	23.52 ± 3.36	25.80 ± 3.34	<0.001
TG^a^, mmol/L	1.00 (0.67–1.55)	0.75 (0.53–1.15)	1.18 (0.77–1.76)	<0.001
TC, mmol/L	4.98 ± 0.98	4.91 ± 0.98	5.01 ± 0.97	0.021
HDL-C, mmol/L	1.22 ± 0.33	1.32 ± 0.37	1.16 ± 0.29	<0.001
LDL-C, mmol/L	2.77 ± 0.96	2.71 ± 0.94	2.79 ± 0.98	0.050
SBP, mmHg	130.26 ± 19.60	123.73 ± 18.52	133.54 ± 19.31	<0.001
DBP, mmHg	82.45 ± 13.05	77.28 ± 11.01	85.05 ± 13.23	<0.001
FBG, mmol/L	5.31 ± 1.30	5.07 ± 0.95	5.43 ± 1.20	<0.001
*MTHFR* C677T				
CC	485 (21.67)	165 (22.00)	320 (21.49)	-
CT	1071 (47.83)	353 (47.07)	718 (48.22)	-
TT	683 (30.50)	232 (30.93)	451 (30.29)	0.875
C allele	2041 (45.58)	683 (45.53)	1358 (45.60)	-
T allele	2437 (54.42)	817 (54.47)	1620 (54.40)	0.966
*MTRR* A66G				
AA	1285 (57.39)	432 (57.60)	853 (57.29)	-
AG	801 (35.77)	265 (35.33)	536 (36.00)	-
GG	153 (6.83)	53 (7.07)	100 (6.72)	0.923
A allele	3371 (75.28)	1129 (75.27)	2242 (75.29)	-
G allele	1107 (24.72)	371 (24.73)	736 (24.71)	0.989

*BMI* body mass index, *TG* triglyceride, *TC* total cholesterol, *HDL-C* high-density lipoprotein cholesterol, *LDL-C* low-density lipoprotein cholesterol, *SBP* systolic blood pressure, *DBP* diastolic blood pressure, *FBG* fasting blood glucose, *MTHFR* methylenetetrahydrofolate reductase, *MTRR* methionine synthase reductase

^a^The value of TG was presented as median (interquartile range), and the difference in serum TG levels was determined by the Wilcoxon two-sample test

The genotype distributions of both the *MTHFR* C677T and *MTRR* A66G polymorphisms were in accordance with Hardy-Weinberg equilibrium (both *P* > 0.05) and were consistent with those previously reported for Tianjin population [35]. The frequency of the *MTHFR* 677CC, CT and TT genotypes was 22.00, 47.07 and 30.93 % in females, and 21.49, 48.22 and 30.29 % in males. As for the *MTRR* A66G polymorphism, the frequency of AA, AG and GG genotypes was 57.60, 35.33 and 7.07 % in women, and 57.29, 36.00 and 6.72 % in men. There was no significant difference between females and males in terms of the genotypic frequencies of the *MTHFR* C677T and *MTRR* A66G polymorphisms (both *P* > 0.05).

### Genotypes and serum lipid levels

For total participants, no significant correlation was found between the *MTHFR* C677T polymorphism and serum lipid levels either in normal weight group or overweight/obesity group (Table 2). When stratified by gender, the *MTHFR* 677TT genotype was associated with higher serum TG levels in female subjects with overweight/obesity (*P* = 0.007). For male subjects with overweight/obesity, the *MTHFR* 677CT genotype carriers had significantly higher LDL-C levels than the *MTHFR* 677TT genotype carriers did (*P* = 0.018). However, in either women or men, no significant difference was detected in the four lipid parameters for normal weight subjects bearing different genotypes of the *MTHFR* C677T polymorphism.

**Table 2:** Interaction of *MTHFR* C677T polymorphism with overweight/obesity on serum lipid levels

BMI	Genotype	*n*	TG (mmol/L)	Lg (TG)	TC (mmol/L)	HDL-C (mmol/L)	LDL-C (mmol/L)
Total							
<24	CC	198	0.71 (0.53–1.07)	−0.10 ± 0.25	4.83 ± 1.07	1.32 ± 0.37	2.65 ± 0.97
	CT	438	0.76 (0.59–1.17)	−0.08 ± 0.24	4.86 ± 1.01	1.31 ± 0.34	2.68 ± 0.95
	TT	249	0.69 (0.49–1.02)	−0.12 ± 0.27	4.80 ± 0.95	1.34 ± 0.36	2.63 ± 0.92
≥ 24	CC	287	1.15 (0.78–1.81)	0.08 ± 0.26^a, b, c^	5.02 ± 0.89	1.17 ± 0.32^a, b, c^	2.79 ± 0.89
	CT	633	1.25 (0.86–1.85)	0.11 ± 0.27^a, b, c^	5.12 ± 0.98^a, b, c^	1.14 ± 0.28^a, b, c^	2.92 ± 1.04^a, b, c^
	TT	434	1.25 (0.86–1.77)	0.11 ± 0.27^a, b, c^	5.02 ± 0.93	1.14 ± 0.28^a, b, c^	2.74 ± 0.91
P* _interaction_			-	0.268	0.668	0.268	0.296
Female							
<24	CC	104	0.65 (0.50–0.89)	−0.16 ± 0.23	4.82 ± 1.03	1.39 ± 0.38	2.66 ± 0.93
	CT	224	0.71 (0.53–0.94)	−0.13 ± 0.24	4.85 ± 0.89	1.36 ± 0.36	2.68 ± 0.93
	TT	142	0.63 (0.44–0.90)	−0.16 ± 0.28	4.82 ± 1.01	1.40 ± 0.39	2.65 ± 0.98
≥ 24	CC	61	0.90 (0.60–1.15)	−0.07 ± 0.22	5.13 ± 0.98	1.28 ± 0.36	2.90 ± 0.87
	CT	129	0.99 (0.65–1.43)	0.00 ± 0.23^a, b, c^	5.04 ± 0.98	1.23 ± 0.31^a, b, c^	2.78 ± 0.90
	TT	90	1.09 (0.78–1.50)	0.05 ± 0.27^a, b, c, d^	4.94 ± 1.10	1.17 ± 0.32^a, b, c^	2.71 ± 1.01
P* _interaction_			-	0.015	0.812	0.161	0.750
Male							
<24	CC	94	0.81 (0.57–1.25)	−0.05 ± 0.25	4.83 ± 1.12	1.24 ± 0.35	2.65 ± 1.01
	CT	214	0.88 (0.65–1.32)	−0.03 ± 0.24	4.87 ± 1.12	1.26 ± 0.30	2.68 ± 0.97
	TT	107	0.79 (0.56–1.18)	−0.07 ± 0.25	4.77 ± 0.88	1.26 ± 0.31	2.60 ± 0.83
≥ 24	CC	226	1.24 (0.87–1.95)	0.12 ± 0.26^a, b, c^	4.99 ± 0.87	1.14 ± 0.30^a, b, c^	2.76 ± 0.89
	CT	504	1.32 (0.91–2.01)	0.14 ± 0.27^a, b, c^	5.14 ± 0.98^b, c^	1.12 ± 0.27^a, b, c^	2.96 ± 1.07^a, b, c^
	TT	344	1.28 (0.86–1.79)	0.12 ± 0.26^a, b, c^	5.04 ± 0.89	1.13 ± 0.26^a, b, c^	2.74 ± 0.88^e^
P* _interaction_			-	0.625	0.747	0.747	0.399

*BMI* body mass index, *MTHFR* methylenetetrahydrofolate reductase

*Adjusted for age

^a^ *P* < 0.05 in comparison with CC genotype of the BMI < 24 subgroup

^b^ *P* < 0.05 in comparison with CT genotype of the BMI < 24 subgroup

^c^ *P* < 0.05 in comparison with TT genotype of the BMI < 24 subgroup

^d^ *P* < 0.05 in comparison with CC genotype of the BMI ≥ 24 subgroup

^e^ *P* < 0.05 in comparison with CT genotype of the BMI ≥ 24 subgroup

Subjects carrying the *MTRR* 66GG genotype had higher serum HDL-C levels than those with *MTRR* 66AA or AG genotypes in overweight/obese individuals (Table 3, *P* = 0.035 and *P* = 0.084, respectively). When stratified by gender, similar association only exists in female subjects with overweight/obesity.

**Table 3:** Interaction of *MTRR* A66G polymorphism with overweight/obesity on serum lipid levels

BMI	Genotype	*n*	TG (mmol/L)	Lg (TG)	TC (mmol/L)	HDL-C (mmol/L)	LDL-C (mmol/L)
Total							
<24	AA	511	0.73 (0.54–1.10)	−0.10 ± 0.24	4.84 ± 1.01	1.31 ± 0.36	2.68 ± 0.96
	AG	309	0.72 (0.53–1.07)	−0.10 ± 0.27	4.84 ± 1.02	1.35 ± 0.34	2.64 ± 0.93
	GG	65	0.77 (0.59–1.20)	−0.07 ± 0.25	4.82 ± 0.90	1.31 ± 0.36	2.62 ± 0.90
≥ 24	AA	774	1.24 (0.86–1.87)	0.11 ± 0.28^a, b, c^	5.07 ± 0.93^a, b^	1.14 ± 0.28^a, b, c^	2.84 ± 0.96^b^
	AG	492	1.21 (0.84–1.69)	0.09 ± 0.25^a, b, c^	5.03 ± 0.98	1.14 ± 0.28^a, b, c^	2.81 ± 0.99
	GG	88	1.21 (0.77–2.14)	0.11 ± 0.27^a, b, c^	5.22 ± 0.95	1.25 ± 0.33^d, e^	2.95 ± 1.02
P*_ interaction_			-	0.438	0.688	0.050	0.608
Female							
<24	AA	277	0.69 (0.49–0.94)	−0.15 ± 0.24	4.80 ± 0.97	1.37 ± 0.38	2.65 ± 0.97
	AG	158	0.66 (0.48–0.90)	−0.15 ± 0.27	4.88 ± 0.93	1.39 ± 0.36	2.70 ± 0.91
	GG	35	0.68 (0.52–0.89)	−0.14 ± 0.22	4.88 ± 1.00	1.39 ± 0.41	2.64 ± 0.90
≥ 24	AA	155	0.96 (0.65–1.41)	−0.00 ± 0.25^a, b^	5.01 ± 0.97	1.22 ± 0.31^a, b^	2.72 ± 0.90
	AG	107	1.00 (0.70–1.46)	0.01 ± 0.25^a, b^	5.01 ± 1.07	1.19 ± 0.31^a, b, c^	2.84 ± 0.96
	GG	18	0.96 (0.57–1.19)	−0.02 ± 0.27	5.31 ± 1.12	1.46 ± 0.50^f^	3.03 ± 1.01
P* _interaction_			-	0.598	0.937	0.056	0.593
Male							
<24	AA	234	0.83 (0.63–1.24)	−0.05 ± 0.23	4.88 ± 1.06	1.23 ± 0.32	2.71 ± 0.95
	AG	151	0.82 (0.57–1.25)	−0.06 ± 0.25	4.79 ± 1.11	1.29 ± 0.31	2.57 ± 0.95
	GG	30	1.02 (0.63–1.47)	0.02 ± 0.26	4.75 ± 0.80	1.22 ± 0.28	2.60 ± 0.92
≥ 24	AA	619	1.30 (0.90–1.97)	0.14 ± 0.27^a, b^	5.09 ± 0.92^b^	1.12 ± 0.27^a, b^	2.87 ± 0.97^b^
	AG	385	1.27 (0.87–1.78)	0.11 ± 0.25^a, b^	5.04 ± 0.95	1.13 ± 0.28^a, b^	2.81 ± 1.00
	GG	70	1.32 (0.82–2.20)	0.14 ± 0.26^a, b^	5.20 ± 0.91	1.19 ± 0.25	2.93 ± 1.03
P* _interaction_			-	0.430	0.606	0.109	0.649

*BMI* body mass index, *MTRR* methionine synthase reductase

*Adjusted for age

^a^ *P* < 0.05 in comparison with AA genotype of the BMI < 24 subgroup

^b^ *P* < 0.05 in comparison with AG genotype of the BMI < 24 subgroup

^c^ *P* < 0.05 in comparison with GG genotype of the BMI < 24 subgroup

^d^ *P* < 0.05 in comparison with AA genotype of the BMI ≥ 24 subgroup

^e^ *P* = 0.084 in comparison with AG genotype of the BMI ≥ 24 subgroup

^f^ *P* = 0.058 in comparison with AG genotype of the BMI ≥ 24 subgroup

### Overweight/obesity and serum lipid levels

As listed in Table 2, overweight/obesity group had higher TG and lower HDL-C levels as compared with normal weight group in *MTHFR* 677CC, CT and TT genotype carriers (All *P* < 0.05), whereas significant difference of TC and LDL-C levels between the two groups were only found in subjects with *MTHFR* 677CT genotype (Both *P* < 0.05). Similarly, overweight/obesity group had higher TG levels than normal weight group in *MTRR* 66AA, AG and GG genotype carriers (Table 3, All *P* < 0.05). The correlation between overweight/obesity and higher TC levels was only found in subjects with *MTRR* 66AA genotype (*P* < 0.05). HDL-C levels were lower in *MTRR* 66AA and AG genotypes carriers with overweight/obesity than in those with normal weight (Both *P* < 0.05). Stratified analyses by gender showed similar results (Tables 2 and 3).

### Interactions of polymorphism with overweight/obesity on serum lipid levels

Participants were divided into six groups according to their genotypes and BMI. Then we analyzed the interaction and compared the difference of serum lipid levels among six groups.

In total population, no significant interaction between the *MTHFR* C677T polymorphism and overweight/obesity on lipid traits was observed (Table 2). However, this polymorphism interacts with overweight/obesity to influence serum TG levels among female subjects (*P* = 0.015). The *MTHFR* 677TT genotype carriers with overweight/obesity had higher serum TG levels than other groups; the differences were statistically significant except for that between the *MTHFR* 677TT genotype carriers with overweight/obesity and the *MTHFR* 677CT genotype carriers with overweight/obesity.

For the *MTRR* A66G polymorphism, we detected a borderline significant interaction between this polymorphism and overweight/obesity on serum HDL-C levels (*P* = 0.050, Table 3). When stratified by gender, this borderline significant interaction only existed in females (*P* = 0.056). The *MTRR* 66AG genotype carriers with overweight/obesity had lower serum HDL-C levels than the others; the differences were statistically significant except for that between the *MTRR* 66AG genotype carriers with overweight/obesity and the *MTRR* 66AA genotype carriers with overweight/obesity.

## Discussion

In the current study, interaction between the *MTHFR* C677T polymorphism and overweight/obesity on serum TG levels, and interaction between the *MTRR* A66G polymorphism and overweight/obesity on serum HDL-C levels were detected in women but not in men. We found that the *MTHFR* 677TT genotype had detrimental effects on serum TG levels and the *MTRR* 66GG genotype had favourable effects on serum HDL-C levels in female subjects with overweight/obesity. Furthermore, in male subjects with overweight/obesity, the *MTHFR* 677CT genotype carriers had higher LDL-C levels than TT genotype.

Many previous studies have explored the relationship between the *MTHFR* C677T polymorphism and serum or plasma lipid profiles in humans, with some indicating that the T allele was associated with unfavourable lipid profiles [8–11, 17, 36–39], whereas the others reported null association [40–43]. An interesting study by Zhang et al. indicated that the *MTHFR* 677 T allele significantly increases the serum levels of TC and LDL-C in both Chinese Bai Ku Yao and Han populations [10]. Among Polish elderly women, the *MTHFR* 677CT/TT genotypes were associated with higher TC and lower HDL-C levels as compared with CC genotype [36]. According to Huang et al. [39], hyperlipidemia patients with *MTHFR* 677CT/TT genotypes had higher TG concentrations than those with CC genotype. However, Li and coworkers failed to find any significant correlations between this polymorphism and four common lipid traits (TG, TC, HDL-C and LDL-C) in hypertensive patients [42]. Various factors including gender may modulate the association between this polymorphism and lipid levels and then lead to inconsistency among these studies.

For instance, Chen et al. initially failed to find significant difference in the four lipid measures (TG, TC, HDL-C and LDL-C) among the *MTHFR* C677T genotypes in longevous individuals [37]. However, when gender was taken into consideration, the *MTHFR* 677 T allele carriers of females but not males were found to have significantly higher levels of TC and LDL-C than CC genotype. Similar finding was also reported in essential hypertensive patients [11]. That study found that female but not male hypertensive patients with *MTHFR* 677TT genotype had higher LDL-C levels than those with CC genotype. In contrast, Zhang et al. found that significant association between serum LDL-C levels and this polymorphism only exists in males in both Chinese Bai Ku Yao and Han populations [10]. In agreement with the findings of Zhang et al., our results indicated that significant correlation between this variant and serum LDL-C levels only existed in males with overweight/obesity. In addition, we also found that *MTHFR* 677TT genotype was associated with higher serum TG levels in females with overweight/obesity. Though heterogeneity by sex for lipid levels had been previously reported for other SNPs [33], the underlying mechanisms for the gender-specific association are unclear. It may be partly resulted from hormone action in combination with social and behavioral differences between men and women [29].

Regarding the *MTRR* A66G polymorphism, our results indicated that *MTRR* 66GG genotype was associated with higher HDL-C levels in female subjects with overweight/obesity. Jiang et al. reported that the serum TC and LDL-C levels were lower in *MTRR* 66GG genotype than in *MTRR* 66AA genotype among Chinese hypertensive patients [11]. Similarly, when stratified by gender, these correlations were only evident in women. But in another sample of Chinese hypertensive patients, a marginally significant association was found between *MTRR* 66GG genotype and elevated serum TC levels [42]. Our group previously conducted a case-control study to investigate the association of this genetic variant with metabolic syndrome [44], and found that *MTRR* 66GG genotype was associated with increased risk of high TG (TG ≥ 1.7 mmol/L), while no significant relationship was detected between this polymorphism and low HDL-C (HDL-C < 1.03 mmol/L for males and < 1.30 mmol/L for females). However, Misiak et al. found there was no significant relationship between this polymorphism and TG or HDL-C levels in both first-episode schizophrenia patients and healthy controls [45]. The gender-specific effect of the *MTRR* A66G polymorphism on blood lipid profiles was not well explored. Moreover, unlike our study selecting general population without any severe diseases, the studies mentioned above were conducted in specific populations with various disorders such as hypertension [11, 42], metabolic syndrome [44] and schizophrenia [45], which may be a crucial contributor for the difference between our results and the prior findings.

In addition, nutrition and dietary habits are also important factors which may modulate the relationship between the two polymorphisms and lipid profiles. It has been reported that many nutraceuticals are able to actively influence lipid profiles and effectively improve dyslipidemia. Furthermore, because of the ability to attack several biochemical pathways of lipid metabolism, nutraceuticals appear to have the potential to overcome the genetic variability of individuals [46]. Thus, future studies should take nutraceuticals into consideration when evaluating the influence of genetics on lipid levels.

Literature about interactions between the two studied polymorphisms and overweight/obesity on blood lipid profiles was limited. In a systematic Medline search, we only found one study that examined interaction between the *MTHFR* C677T polymorphism and overweight/obesity on serum lipid levels [5]. In that study, Yin et al. observed that the *MTHFR* C677T polymorphism interacted with high BMI to influence serum levels of TG, TC and LDL-C in Chinese Bai Ku Yao population. We failed to find significant interaction between the *MTHFR* C677T polymorphism and overweight/obesity, whereas a borderline significant interaction between the *MTRR* A66G polymorphism and overweight/obesity on serum HDL-C levels was observed.

Considering the gender differences in lipid and lipoprotein metabolism, we further assessed whether the interactions exhibit gender-specificity. Then we found that the *MTHFR* 677TT genotype interacts with overweight/obesity to increase serum TG levels in females, a finding consistent with the result from Chinese Bai Ku Yao population [5]. Likewise, the borderline significant interaction between the *MTRR* A66G polymorphism and overweight/obesity was only evident in women. It is difficult to compare our findings with other studies, because, to the best of our knowledge, this is the first study reporting gender-specific interactions of the two polymorphisms with overweight/obesity on serum lipid levels. Although many studies have reported that certain lipid-related genetic variants interact with environmental factors, such as high BMI, alcohol consumption and cigarette smoking, to modify blood lipid profiles [7, 47–50], all of them were performed on women and men combined. If our gender-specific findings reflect true causal associations, it would indicate that women with the *MTHFR* 677TT or the *MTRR* 66AG genotypes are more susceptible to the negative influence from overweight/obesity on some lipid traits. Future work is needed to clarify the underlying mechanism and confirm or refute our findings.

In this study, we selected ethnically homogeneous population (Chinese Han), which would minimize population stratification bias. Despite the assets of the current study, several limitations should be considered. First of all, although the sample size was relatively large, the number of female subjects in some subgroup analyses was a bit small. Next, the limitation of generalization of our results was that we enrolled the study subjects from one hospital. However, genotype distributions of the two polymorphisms were in accordance with Hardy-Weinberg equilibrium and were also consistent with those previously reported for Tianjin population [35]. The evidence suggests that our study subjects still have certain representativeness. Finally, it is well known that serum lipid levels are influenced by both genetic and environmental factors as well as their interactions. We only investigated two polymorphisms and did not have data on other environmental factors, such as dietary habits, alcohol consumption and cigarette smoking. Thus, the role of other genes, other environmental exposures and other gene-environment interactions on serum lipid profiles remain to be determined.

## Conclusions

In conclusion, we present the first study to date on the gender difference in the interactions of the *MTHFR* C677T and *MTRR* A66G polymorphisms with overweight/obesity on some serum lipid traits. Interaction between the *MTHFR* C677T polymorphism and overweight/obesity on TG levels was only evident in women, as was interaction between the *MTRR* A66G polymorphism and overweight/obesity on HDL-C levels. In the future, well-designed studies are required to confirm or refute our findings.

## Abbreviations

BMI: Body mass index
DBP: Diastolic blood pressure
FBG: Fasting blood glucose
HDL-C: High-density lipoprotein cholesterol
LDL-C: Low-density lipoprotein cholesterol
MTHFR: Methylenetetrahydrofolate reductase
MTRR: Methionine synthase reductase
SBP: Systolic blood pressure
TC: Total cholesterol
TG: Triglyceride
